# Taxonomic Revision of Hook-Forming *Acrosorium* (Delesseriaceae, Rhodophyta) from the Northwestern Pacific Based on Morphology and Molecular Data

**DOI:** 10.3390/plants10112269

**Published:** 2021-10-22

**Authors:** Jeong Chan Kang, Showe-Mei Lin, Kathy Ann Miller, Myung Sook Kim

**Affiliations:** 1 Research Institute for Basic Sciences, Jeju National University, Jeju 63243, Korea; mantachan@hanmail.net; 2 Institute of Marine Biology, National Taiwan Ocean University, Keelung 20224, Taiwan; linsm@ntou.edu.tw; 3 Silva Center for Phycological Documentation, University Herbarium, University of California, Berkeley, CA 94720, USA; kathyannmiller@berkeley.edu; 4 Department of Biology, Jeju National University, Jeju 63243, Korea

**Keywords:** *Acrosorium*, Delesseriaceae, hook-forming thalli, molecular phylogeny, morphology, taxonomy

## Abstract

Cosmopolitan *Acrosorium* species with hook-forming thalli have been merged under the name of *Acrosorium ciliolatum* (Harvey) Kylin through a long and complicated nomenclatural history. We examined the specimens of ‘*A. ciliolatum’* and related taxa from the northwestern (NW) Pacific, the UK, southern Spain, Australia, New Zealand, and Chile, using morphological and molecular analyses. We confirmed that these specimens are separated into four clades based on *rbc*L phylogeny, and the absence or presence of terminal hook-like structures represent intraspecific variation. Our results indicated that *Acrosorium flabellatum* Yamada, *Cryptopleura hayamensis* Yamada, *Cryptopleura membranacea* Yamada and the entities known as ‘*A. ciliolatum’* in the NW Pacific are conspecific; the name *A. flabellatum* is the oldest and has priority. This taxon exhibits extreme variations in external blade morphology. We also confirmed that the position of the tetrasporangial sori is a valuable diagnostic characteristic for distinguishing *A. flabellatum* in the NW Pacific. We also discussed the need for further study of European and southern hemisphere specimens from type localities, as well as the ambiguous position of California specimens.

## 1. Introduction

The cosmopolitan genus *Acrosorium* Zanardini ex Kützing [1] currently comprises 11 species [2]. *Acrosorium ciliolatum* (Harvey) Kylin [3], with its distinctive hook-forming branches [4,5,6], is the most widely distributed species, occurring from tropical to subpolar regions in both the southern and northern hemispheres [2]. This species was described by Harvey in 1855 as *Nitophyllum ciliolatum* on the basis of specimens from King George Sound, Western Australia and Tasmania [7]. Two other species have a part in the nomenclatural history of *A. ciliolatum*: *Nitophyllum venulosum* Zanardini was described from Zara, Croatia, Adriatic Sea in 1866 [8] and transferred to *Acrosorium* by Kylin as *Acrosorium venulosum* (Zanardini) Kylin in 1924 [3]; and *Fucus laceratus* var. *uncinatus* Turner 1808 [9] (type locality, Devonshire, UK) was elevated to species-level and transferred to *Acrosorium* by Kylin, also in 1924 [3]. Wynne [6] proposed that *A. venulosum* is the correct name for the globally distributed hook-forming *Acrosorium* species going under the name of *A. uncinatum*, and authentic *A. uncinatum* is conspecific with *Cryptopleura ramosa* (Hudson) L. Newton [10]. Womersley merged *A. venulosum* with *A. ciliolatum* in 2003 [5]. Currently, the name *Acrosorium ciliolatum* is applied to species with hook-forming thalli [2,5,11,12,13,14,15,16,17].

In the northwestern (NW) Pacific, a hook-forming *Acrosorium* species was first reported by Okamura [18] as *Nitophyllum uncinatum* (Turner) J. Agardh; this entity was later identified as *A. ciliolatum* [19,20,21] or *A. venulosum* [22,23,24]. Subsequently, related species *Acrosorium flabellatum* Yamad [25], *Acrosorium yendoi* Yamada [25], *Cryptopleura membranacea* Yamada [26], *Acrosorium polyneurum* Okamura [27], *Cryptopleura hayamensis* Yamada [28], and *Acrosorium okamurae* Noda in [29], were described. *A. ciliolatum* (or *A. venulosum*) in the NW Pacific, *A. flabellatum*, *C. membranacea*, and *C. hayamensis* have tetrasporangial sori restricted to marginal branchlets [23,25,26,27,30]. To date, morphological features, such as thallus habit, branching pattern, apex shape, hook formation, and the presence or absence of basal macroscopic veins, have been used to distinguish these four species [20,23,31]. 

**Figure 1:**
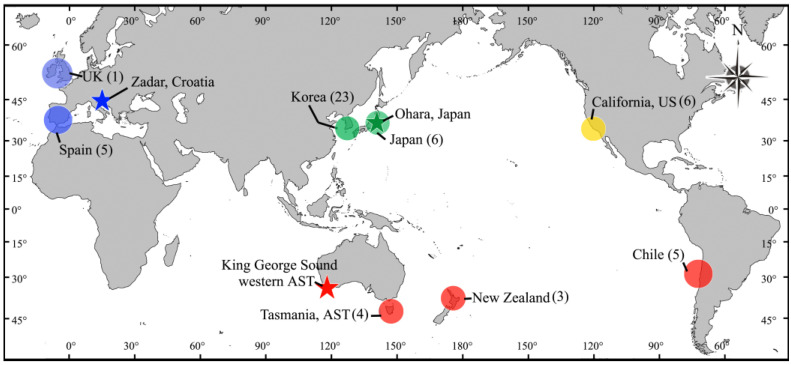
A map showing the sampling locations (circles: blue from Europe; green from NW Pacific; yellow from California; red from southern hemisphere), and type localities for *A. venulosum* (blue star), *A. flabellatum* (green star), and *A. ciliolatum* (red star). Numbers: sample number for molecular analysis.

During an investigation of species and genetic diversity in the family Delesseriaceae from the NW Pacific, we encountered difficulties in identifying specimens of these four species, due to overlapping morphological features. To resolve this problem, we attempted to identify meaningful characters delimitating the four species, based on morphology and *rbc*L sequences. Remarkably, our molecular phylogeny showed that the four species are conspecific, with *A. flabellatum* having priority as the earliest name. To determine the relationship of this *Acrosorium/Cryptopleura* complex in the NW Pacific to specimens identified as *A. ciliolatum* in other parts of the world, we obtained specimens from the UK, southern Spain, southern Australia, New Zealand, Chile, and California, USA, and compared their morphological and molecular characteristics (Figure 1). 

## 2. Results

### 2.1. Molecular Analyses

The phylogenetic analyses of *rbc*L included 1443 bp, of which 412 bp (28.6%) were variable and 434 bp (17.9%) were phylogenetically informative. The *rbc*L sequences in the NW Pacific *Acrosorium*/*Cryptopleura* complex differed by 0.73 to 0.98% from Californian, 1.55 to 2.05% from European (Spain and UK), and 1.31 to 1.55% from southern hemisphere (Chile, New Zealand, and Tasmania) samples. The sequences from Japan and Korea varied by up to 0.32%.

**Figure 2:**
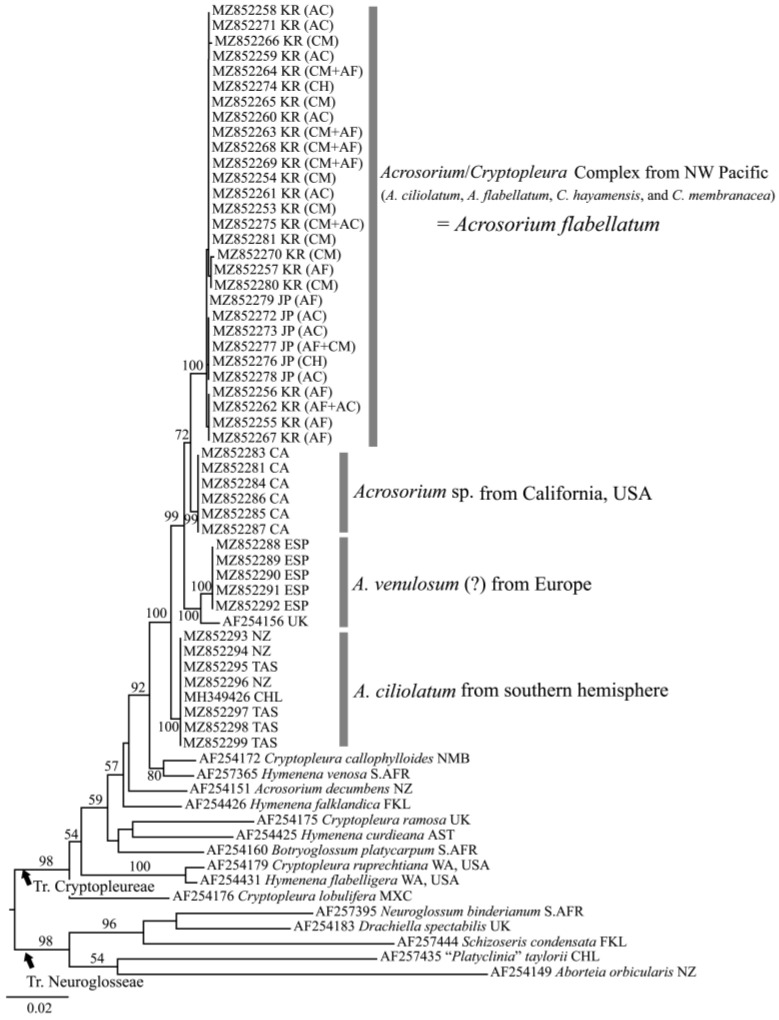
Maximum likelihood phylogenetic tree for the tribe Cryptopleureae derived from plastid-encoded *rbcL* sequence data. Bootstrap values (1000 replicates) are shown above branches. For NW Pacific samples, letters in parentheses refer to phenotype classifications: AC, *Acrosorium ciliolatum*; AF, A. *flabellatum*; CH, *Cryptopleura hayamensis*; CM, *C. membranacea*; +, overlapping phenotype. Scale bar represents substitutions per site.

**Figure 3:**
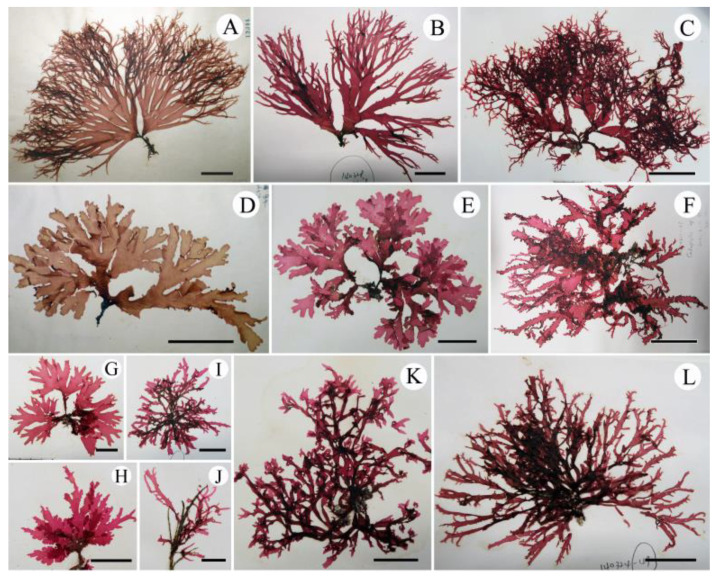
*Acrosorium flabellatum* Yamada. (**A**) Holotype specimens (SAP 12344) of *A. flabellatum*. (**B**) JN1403280085 from the type locality, Enoshima of Japan, phenotype as AF. (**C**) JN1307310149 from Yeosu of Korea, as AF + AC. (**D**) SAP 15293, holotype specimens of *C. membranacea* Yamada. (**E**) JN1404160015 from Wando of Korea, as CM. (**F**) JN1106110035 from Udo of Korea, as AC + CM. (**G**) JN1306040012 from Chujado of Korea, as AF + CM. (**H**) JN1304050003 from Jeju of Korea, as CM + AC. (**I**–**K**) JN1403280017 from Shimoda of Japan, JN1303260008 from Jeju of Korea, and JN1308020036 from Wando of Korea, respectively, phenotype as AC. (**L**) JN1403240049 from Chiba of Japan, as CH. (Phenotype: AC, *A. ciliolatum*; AF, *A. flabellatum*; CH, *C. hayamensis*; CM, *C. membranacea*; +, overlapping phenotype). Scale bars: (**C**,**D**–**F**), 5 cm; (**A**,**L**), 3 cm; (**B**,**E**,**G**–**I**,**K**), 2 cm; (**J**), 1 cm.

In the phylogenetic tree based on *rbc*L gene (Figure 2), the sequences from the NW Pacific (Japan and Korea), California, Europe, and southern hemisphere formed independent and monophyletic clades with high bootstrap values (100, 99, 100, and 100%, respectively) in the tribe Cryptopleureae. The clades of the NW Pacific and California formed sister-groups, with a low bootstrap value (72%). In the NW Pacific clade, the *rbc*L sequences from each phenotype—AF (Figure 3B), CM (Figure 3E), AC (Figure 3I,J), and CH (Figure 3L)—and the overlapping phenotypes (Figure 3C,F–H) were all in the same clade without any distinct structure (Figure 2).

### 2.2. Morphological Observations

#### 2.2.1. *Acrosorium flabellatum* Yamada 1930:31

**Figure 4:**
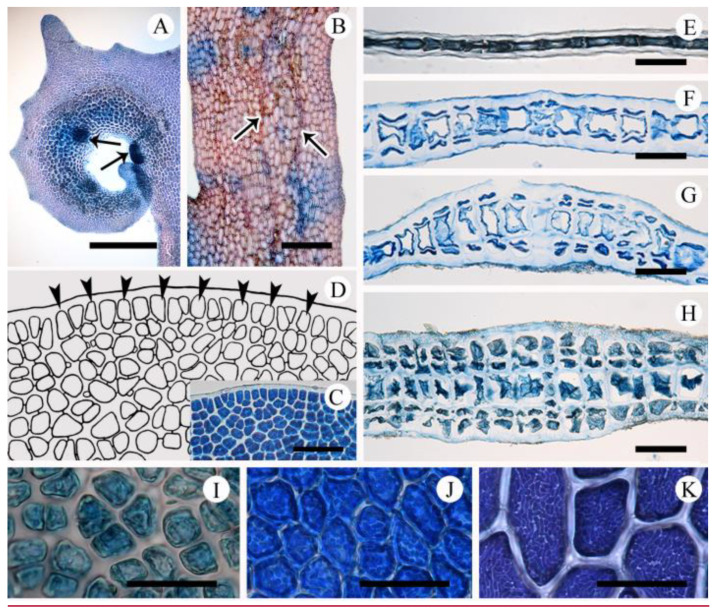
Vegetative structures of *Acrosorium flabellatum* Yamada. (**A**) Terminal hook with multi-cellular peg-like rhizoids (arrows). (**B**) Surface view of middle part of thallus with microscopic veins (arrows). (**C**,**D**) Apex of young thallus showing numerous obliquely dividing apical cells (arrowheads). (**E**–**H**) Cross-sections of blade through upper (**E**), middle (**F**), stipe (**G**), and basal (**H**) portions of blade. (**I**–**K**) Polygonal cortical cells with parietal chloroplasts from near meristematic (**I**), middle (**J**) and lower (**K**) parts of blade. Scale bars: (**A**), 1 mm; (**B**), 300 µm; (**E**–**H**), 100 µm; (**C**,**J**,**K**), 50 µm; (**I**), 30 µm.

**Figure 5:**
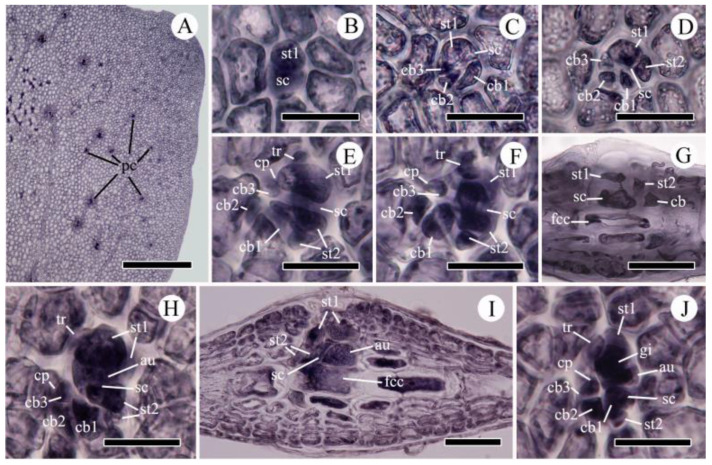
Female reproductive structures of *Acrosorium flabellatum* Yamada. (**A**) Positions of procarps (pc). (**B**–**G**) Procarp development. (**G**) Cross-sectioned mature procarp. (**H**) Surface view of early post-fertilization stage. (**I**) Cross-section view of early post-fertilization stage. (**J**) Surface view of young cystocarp before pericarp formation. au, auxiliary cell; cb, carpogonial branch (numbers, cell order); cp, carpogonium; fcc, fertile central cell; gi, gonimoblast initial; sc, supporting cell; st1, first sterile cell group; st2, second sterile cell group; tr, trichogyne. Scale bars: (**A**), 500 µm; (**C**−**I**), 50 µm; (**J**), 30 µm.

- Specimens examined (Figure 3, Figure 4 and Figure 5)

From Korea: Udo, Jeju (⊕, ♀, 11 June 2011); Jeongdori, Wando (9 June 2012); Biyangdo, Jeju (⊕, ♀, ♂, 17 February 2013,); Jongdal, Jeju (3 February 2013); Gapado, Jeju (♀, ♂, 4 March 2013; ⊕, ♀, ♂ 26 March 2013); Seopseom, Jeju (5 April 2013); Gangjeong, Jeju (⊕, 22, May 2013); Chujado, Jeju (4 June 2013); Gyeongpo, Gangreung (27 October 2012); Songjeong, Busan (20 December 2012); Dokdo, Ulleung (22 April 2013); Jukbyeon, Uljin (19 July 2013); Geomundo, Yeosu (31 July 2013); from Japan: Chiba (9 April 2013); Gochome, Shimoda (⊕, 12 April 2013; 26 March 2014), Enoshima, Kanagawa (28 March 2014); Ohara, Chiba (24 March 2014); Yoshio, Chiba (23 March 2014), Toji, Shimoda (28 March 2014).

- Vegetative morphology (Figure 3 and Figure 4)

Thalli are extremely variable in shape and size, epilithic or epiphytic, membranous, up to 30 cm high, pinkish red when alive, turning brownish red when dry, each consisting of single to several large erect blades and a small prostrate base. When growing on rock or concrete, the erect blades expand from a compressed short stipe and are divided palmately or di- to trichotomously several times, broadening into a flabellate outline (Figure 3A–H). When growing on other algae such as *Corallina* spp. or *Cladophora* spp., the erect blades are usually linear and ribbon-shaped without a distinct stipe, and branched in alternate, di- to trichotomous or irregular pattern, forming tangled clumps (Figure 3I–L). Blade margins are entire with small semi-circular proliferations that occasionally elongate to form marginal branches (Figure 3A–C,F,I–L). Blades tips are round (Figure 3D–F,H) to acute with terminal curves (Figure 3A,B,H,L), hooks (Figure 3C,I–K), or not (Figure 3D–G). Number and degree of hooks are variable (Figure 3A–L).

Multicellular peg-like rhizoids are often observed along the inner surfaces of terminal hooks (Figure 4A). Microscopic veins are one cell thick, and extend distally (Figure 4B). Macroscopic veins are observed in the basal parts of thalli when the stipe is well-developed (Figure 3A–H and Figure 4G,H), otherwise not (Figure 3I–L). Apical growth is via numerous obliquely dividing apical meristematic cells, followed by intercalary cell division (Figure 4C,D). Erect blades are mostly monostromatic, except microscopic veins, stipes, and lower portions of the thallus (Figure 4E–H). In cross-section, cortical cells are cut off periclinally from large tetragonal central cells (Figure 4G,H). Cortical cells in the blade are polygonal with parietal chloroplasts. Chloroplasts are lobed in young cells, soon becoming numerous and discoid, then elongating in mature cells (Figure 4I–K).

**Figure 6:**
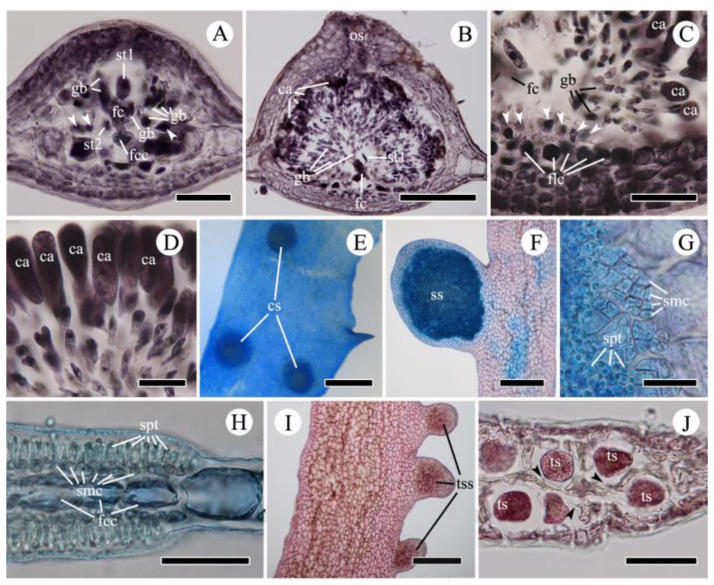
Reproductive structures of *Acrosorium flabellatum* Yamada. (**A**,**B**) Cross-sectioned young (**A**) and mature (**B**) cystocarp. (**C**) Base of cystocarp (arrow heads: small cells from floor cells). (**D**) Carposporangia. (**E**) Surface view of mature cystocarp. (**F**) Spermatangial sorus. (**G**,**H**) Surface (**G**) and cross-sectional views (**H**) of spermatangial sorus. (**I**) Tetrasporangial sori. (**J**) Cross-sectioned tetrasporangial sorus (arrowheads: pit-connection). ca, carposporangium; cs, cystocarp; fc, fusion cell; fcc, fertile central cell; flc, floor cell; gb, gonimoblast cell; smc, spermatangial mother cell; spt, spermatangium; ss, spermatangial sorus; st1, first sterile cell group; st2, second sterile cell group; tss, tetrasporangial sorus; ts, tetrasporangium. Scale bars: (**E**), 1 mm; (**F**,**I**), 500 µm; (**B**,**J**), 300 µm; (**A**,**C**), 100 µm; (**D**,**H**), 50 µm; (**G**), 30 µm.

- Reproductive morphology (Figure 5 and Figure 6)

Gametophytes are dioecious. Procarps are scattered on both sides of the thallus surface, usually along microscopic veins near meristemic portions (Figure 5A). Formation of the procarp is initiated by cutting off a supporting cell from the fertile central cell. The supporting cell initially cuts off the first sterile-cell group, then the carpogonial branch initial which forms the carpogonial branch by sequential division (Figure 5B‒F). When the carpogonial branch is three-celled, the supporting cell cuts off a second sterile-cell group initial which usually divides once before fertilization (Figure 5C‒F). The mature procarp consists of a supporting cell, a four-celled carpogonial branch with a terminal trichogyne swollen at the tip, a one- to two-celled first sterile-cell group, and, usually, a two-celled second sterile-cell group (Figure 5E,F). Procarps develop on both sides of the blade from a common fertile central cell; however, only one procarp develops into the cystocarp (Figure 5G,I).

After presumptive fertilization, the supporting cell is enlarged and distally cuts off an auxiliary cell which divides to form a gonimoblast cell initial (Figure 5H–J). Cell fusions among the fertile central cell, supporting cell, and auxiliary cell occur in an early stage of cystocarp development, during which the gonimoblast cell initials develop into gonimoblast filaments by sequential cell division (Figure 6A). Neither sterile-cell group participates in the formation of the fusion cell, but they remain around the fusion cell (Figure 6A,B). As the cystocarp grows, the fusion cell enlarges by incorporating neighboring cells in the cystocarp floor. Some floor cells cut off one to two layers of small cells in the direction of the cystocarp cavity (Figure 6B,C). The gonimoblast filaments extend radially by cell division with subdichotomous or alternate branching, producing a single carposporangium on each terminal cell (Figure 6B–D). The carposporangia are ovoid to elliptical, 60‒80 µm long, 25‒30 µm wide (Figure 6D). Mature cystocarps are hemispherical with a protruding ostiole (Figure 6B,E). 

Spermatangial sori are produced on both surfaces of small marginal proliferations (Figure 6F). Spermatangial mother cell initials are cut off fertile central cells by periclinal divisions on both blade surfaces. The initial cells divide anticlinally several times to form spermatangial mother cells, all of which cut off one to two clavate spermatangia (Figure 6G,H). 

Tetrasporangial sori are formed on small marginal proliferations (Figure 6I). The tetrasporangia are globose, 70‒100 µm in diameter, and tetrahedrally divided. They are usually cut off from central cells, or, rarely, from inner cortical cells, and arranged in two layers on opposite sides of the central cells (Figure 6J).

#### 2.2.2. *Acrosorium ciliolatum* from Beyond the NW Pacific

**Figure 7:**
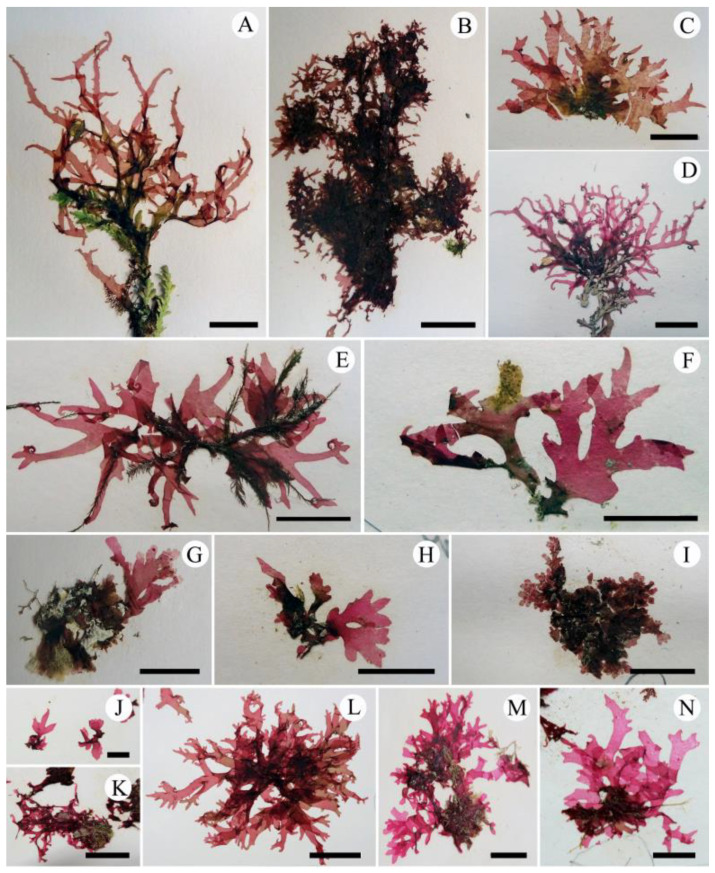
Specimens of ‘*Acrosorium ciliolatum’* from outside of the NW Pacific. (**A**–**C**) From Tasmania, Australia ((**A**), JN15110800R5; (**B**), JN1309080014; (**C**), JN1511080067). (**D**) From New Zealand (JN1309080014). (**E**–**I**) From Spain ((**E**), JN1404280001; (**F**), JN1404300010; (**G**), JN1404280053; (**H**), JN1404280046; (**I**), 1405010034). (**J**–**N**) from California, USA ((**J**), UC1934340; (**K**), UC1965235; (**L**), UC1835679; (**M**), UC2034311; (**N**), UC2034278). Scale bars: (**A**–**J**,**M**,**N**), 1 cm; (**K**–**L**), 2 cm.

- Specimens examined (Figure 7)

From southern hemisphere. Tasmania, Australia: Eaglehawk Neck (⊕, 8 November 2015); New Zealand: Muritai, East Bourne (14 September 2013); Cape Palliser, Ngawi (8 September 2013); Mathesons Bay, North Auckland (27 September 2013).

From Europe. Spain: Roche, Cádiz (29 April 2014); Atlanterra, Tarifa (30 April 2014); De Valdevatueros, Tarifa (⊕, 1 May 2014); La Caleta, Cádiz (28 April 2014). 

From California, USA. South end of Monterey Bay, Monterey County (2 June 1967); Diablo Canyon, San Luis Obisbo County (16 December 2008); Santa Cruz Island, Santa Barbara County (14 September 2007, 14 September 2005); Dana Point, Orange County (12 December 2012); Santa Barbara Island, Santa Barbara County (12 September 2005).

- Habit (Figure 7)

From southern hemisphere (Figure 7A–D): Thalli are brownish to pinkish red, membranous and epilithic or epiphytic on other algae, usually coralline algae. Several erect blades arise from the margins of a small prostrate base, forming tangled clumps. Erect blades are linear with cuneate bases and acute apexes, measure up to 5 cm high and less than 3 mm wide, and are branched in an alternate to irregular pattern, broadening to a flabellate outline (Figure 7A–D). Margins of erect blades are entire. When growing on other macroalgae, blade tips usually terminate in hooks, with small proliferations along blade margins (Figure 7A,B,D). When growing on rock surfaces, hook-shaped tips are absent (Figure 7C). 

From southern Europe (Figure 7E–I): Thalli are pinkish red, membranous and epilithic or epiphytic on other algae. Fronds consist of small basal prostrate blades and erect upper flabellate blades. Erect blades are alternate, palmate, or di- to trichotomous, with cuneate bases and entire margins, up to 2 cm tall, and less than 5 mm wide (Figure 7E–I). Thallus tips are acute with terminal hooks (Figure 7E) when epiphytic on other algae, or acute (Figure 7F) or rounded without terminal hooks (Figure 7G–I) when attached to bedrock.

From California (Figure 7J–M): Thalli are pinkish to brownish red and membranous. Fronds consist of small basal prostrate blades and erect flabellate blades. Erect blades are usually ribbon-shaped, some cuneate, with alternate, or di- to trichotomous branches, up to 7 cm tall, and less than 5 mm wide (Figure 7J,K). Thallus margins are entire with marginal semi-circular proliferations (Figure 7K–N). Thallus tips are acute (Figure 7J–L) to blunt (Figure 7M,N), with terminal hooks (Figure 7K,L) or not (Figure 7J,M,N).

## 3. Discussion

The hook-forming *Acrosorium* species, currently known as *A. ciliolatum*, have a long and complicated nomenclatural history. After the description of *Nitophyllum ciliolatum* in 1855 by Harvey (type locality King George Sound, Western Australia and Tasmania) [7], *Nitophyllum venulosum* Zanardini (in 1866) [8], and *Acrosorium aglaophylloides* Zanardini ex Kützing (in 1869) [1] were sequentially described from Croatia on the Adriatic Sea. Kylin [3] noticed that *A. aglaophylloides* had been redescribed under the name *N. venulosum*; then, he made the new combination *Acrosorium venulosum* (Zanardini) Kylin, and synonymized *A. aglaophylloides* with *A. venulosum*. In the same study [3], he transferred six more Delesseriacean species to the genus *Acrosorium*, including *Nitophyllum reptans* Crouan & Crouan, *N. acrospermum* J. Agardh, *N. corallinarum* Nott, *N. uncinatum* J. Agardh, *N. ciliolatum* Harvey, and *Cryptopleura minor* Sonder. At that time, Kylin [3] mentioned that *A. venulosum* differed from *A. uncinatum*: the former has a rounded apex, whereas the latter has a hook-shaped one. However, he was not confident about separating the three species, *A. venulosum*, *A. uncinatum*, and *A. reptans*. Wynne [6] re-examined the type specimens and Kylin’s [3] original illustrations of the three *Acrosorium* species and concluded that: (1) the hook-forming thalli are morphologically variable in these taxa, (2) *A. uncinatum* and *A. reptans* are conspecific with *Cryptopleura ramosa* (Hudson) L. Newton, and (3) *A. venulosum* is the correct name for the cosmopolitan *A. uncinatum sensu* Kylin [3]. Womersley [5] mentioned a quotation from Kylin [32], “*A. ciliolatum* is not specifically distinct from *A. venulosum* and is the earliest name for this *Acrosorium*”. He therefore merged *A. venulosum* with *A. ciliolatum*. Thus, *Acrosorium* populations with hook-forming thalli have been identified under the name *A. ciliolatum* [17].

Morphological plasticity, intraspecific variation in form due to environmental differences, makes identifying specimens difficult [33,34,35,36,37]. Similarly, different species with convergent morphologies are difficult to distinguish [38,39,40]. These dilemmas are not confined to any particular taxon or region, but to various macroalgae worldwide. Molecular methods using DNA sequencing have improved the delimitation of species with similar morphologies, as well as species with morphological plasticity in various taxonomic groups [33,37,41,42,43]. In particular, chloroplast-encoded *rbc*L sequences are useful for species-level delimitation among members of the Delesseriaceae (e.g., [37,41,42,44,45,46]), and a large quantity of verified *rbc*L sequences from previous studies is available from GenBank [47].

Our molecular and morphological evidence supports Wynne’s [6] opinion that the presence or absence of hook-forming thalli cannot be used as a morphological trait to distinguish among *Acrosorium* species, because this trait shows intraspecific variation and has arisen more than once in the evolution of *Acrosorium* species. Furthermore, our phylogenetic tree (Figure 2) and specimens (Figure 3 and Figure 7) indicate that there are extensive variations in thallus habit and size, branching pattern, apex shape, and the presence or absence of a basal stipe within each population, in addition to the formation of apical hooks. There is no correlation between the clades of our *rbc*L tree and any of the abovementioned external morphological features (Figure 2, Figure 3 and Figure 7). We also confirmed morphological similarities among specimens from different clades (e.g., Figure 3J vs. Figure 7A,D; Figure 7C vs. Figure 7F; Figure 3H vs. Figure 7G). Uncertainties with respect to species identification arise from intraspecific morphological variations, as well as similarities among species. Consequently, relying only on morphology, it was impossible to distinguish *Acrosorium* specimens from different regions. 

The *rbc*L tree (Figure 2) shows that our samples fell into four clades: three distinct clades (the NW Pacific, Europe, and southern hemisphere) and a less well-supported California clade. We conclude that the name *A. ciliolatum* represents a complex consisting of at least three different species, contrary to the opinions of Wynne [6] (*A. venulosum* = globally known as *A. uncinatum*) and Womersley (*A. ciliolatum* = *A. venulosum*) [5]. We found that the position of the tetrasporangial sori is a conclusive feature for distinguishing the NW Pacific specimens from those from other regions. Taxa from the NW Pacific, including ‘*A. ciliolatum’ auctorum*, *A. flabellatum*, *C. hayamenis*, and *C. membranacea* have tetrasporangial sori restricted to small marginal proliferations (Figure 6I) [20,23,25,26,28,30,48]. Tetrasporangial sori are formed on the thallus tip or on marginal proliferations in European ‘*A. ciliolatum’* [4], but are restricted to the thallus tip in Australian *A. ciliolatum* [5]. 

In the NW Pacific, Okamura [18] first reported this entity as *Nitophyllum uncinatum* (Turner) J. Agardh. Later, he provided a detailed description with many illustrations (pl. XXVI, figures 1–19, [30]). He illustrated several variations in habit, including thalli both with and without hooks, linear or broader, and with round or acute tips, under the name of *N. uncinatum*, and regarded these variations as intraspecific. He also described tetrasporangial sori as produced on small marginal proliferations (pl. XXVI, figures 12 and 13 [30]). Yamada [25,26] then described *Acrosorium flabellatum* from Ohara, Kazusa and Enoshima, Sagami, Japan, and *Cryptopleura membranacea* from Amakusa, Kyushyu, Japan, which resembled Okamura’s [30] flabellate frond with an acute and somewhat bent apex (pl. XXVI, figure 3, [30]), and a palmate frond with a basal stipe and round apex (pl. XXVI, figure 5, [30]), respectively. Yamada [28] described another species, *Cryptopleura hayamensis*, from Hayama, Sagami, Japan, with linear thalli lacking hooks. Noda [in 29] described *Acrosorium okamurae*, which was merged with *A. flabellatum* by Yoshida [23]. Among the various phenotypes of *N. uncinatum sensu* Okamura [30], only the specimens with irregularly branching linear thalli with terminal hooks (pl. XXVI, figure 1, [30]) have been referred to as *A. uncinatum sensu* Kylin, then currently identified as *A. venulosum* or *A. ciliolatum* [19,20,21,22,23,24,31]. 

Although Yamada [25,26,28] established *A. flabellatum*, *C. membranacea*, and *C. hayamensis* on the basis of the various phenotypes of *N. uncinatum sensu* Okamura [30], the phenotypic overlaps (Figure 3C,F–H) among numerous individuals raise the question as to whether these phenotypes are independent species. Our results provide a clear resolution to this problem, strongly supporting Okamura’s [30] opinion that the differences in thalli width, apex shape, and branch pattern are variations within a single species. All specimens from Korea and Japan, with their various phenotypes (i.e., AC, AF, CH, and CM) and overlapping phenotypes (Table S1 and Figure 3A–L), are conspecific (Figure 2). Although analyses using more variable mitochondrial or nuclear markers might reveal population-level differences within this clade, we consider the *rbc*L data sufficient support at the species level. Therefore, we propose the merging of ‘*Acrosorium ciliolatum’ auctorum* in the NW Pacific, *Cryptopleura hayamensis* Yamada, and *Cryptopleura membranacea* Yamada with *A. flabellatum* Yamada, which is the earliest name. This taxon is distinguishable from the European and Australian taxa by *rbc*L sequences and the position of the tetrasporangial sori, which are restricted to marginal proliferations (Figure 6I) [4,5,20,23,27,30,48]. 

In this study, the *rbc*L tree (Figure 2) shows that the European *Acrosorium* clade, which currently goes under the name of *A. ciliolatum*, is clearly separate from the southern hemisphere *A. ciliolatum*. However, we will postpone the formal taxonomic treatment of the European clade until examination of specimens from the two type localities, Zadar, Croatia for the European, and King George Sound, western Australia for the southern hemisphere populations. The Californian specimens form a clade that is sister to the NW Pacific clade, with borderline bootstrap support and genetic differentiation (Figure 2). Moreover, we could not find reproductive structures including tetrasporangial sori or identify any morphological differences from the other clades despite examining more than 50 specimens in the UC herbarium. According to Abbott and Hollenberg [49], fertile fronds are rare and only tetrasporophytes have been reported in California, without any description of the position of the tetrasporangial sori. Further studies using additional and more sensitive markers are essential to probe and clarify the relationship of California *Acrosorium* to its sister clade in the NW Pacific.

Existing studies on red algal phylogeny have focused on the relationships between female reproductive structures and molecular phylogeny. Most of these studies indicate that the developmental patterns of carposporophytes provide important information for delimitating genera in the orders Halymeniales, Gigartinales, and Ceramiales [39,42,44,45,46,50,51,52]. During our collections, male and female gametophytes were rarely collected; most were from the Jeju coast of Korea. Most of our collections, including California specimens, were vegetative or tetrasporic thalli. For this reason, our observations of female reproductive structures were conducted only on *A. flabellatum* from the Korean coast. The female reproductive anatomy of *A. flabellatum* is nearly identical to that of *A. acrospermum* (J. Agardh) Kylin described by Papenfuss [13].

## 4. Taxonomic Conclusions

Acrosorium flabellatum Yamada (p. 31, [25]).

**HOLOTYPE:** SAP 12344 (Figure 3A) deposited in SAP, Hokkaido University, Japan (p. 959, [23]).

**TYPE LOCALITY:** Ohara, Kazusa Prov., Japan (p. 959, [23]).

**SYNONYMS:** *Acrosorium ciliolatum auctorum* in the NW Pacific (p. 132, [19]), (figure 46A–C, [20]), *non A. ciliolatum* (Harvey) Kylin; *Acrosorium okamurae* Noda in (p. 45, figure 12, [29]); *Acrosorium uncinatum auctorum* in NW Pacific (p. 225, [53]), (p. 786, pl. 379, [27]), (p. 315, pl. 67, figure 256, [31]), (p. 113, figures A,B, [48]), (p. 266, figures A–C, [54]), *non A. uncinatum* (Turner) Kylin (p. 78, [3]); *Acrosorium venulosum auctorum* in NW Pacific (p. 960, [23]), (p. 267, figures A–C, [54]), *non A. venulosum* (Zanardini) Kylin (p. 77, [3]); *Cryptopleura hayamensis* Yamada (p. 200, pl. 43, figure 2, [28]); *Cryptopleura membranacea* Yamada (p. 28, pl. 11, figure 1, [26]); *Nitophyllum uncinatum sensu* Okamura (p. 49, [18]), (p. 121, pl. XXVI, [27]), non *N. uncinatum* (Turner) J. Agardh (p. 654, [55]).

## 5. Materials and Methods

### 5.1. Collections

Specimens were collected from the coast of the Korean peninsula, eastern Honshu in Japan, New Zealand, Tasmania in the south-eastern Australia, and the Spanish side of the Gibraltar Strait either intertidally or subtidally by SCUBA diving. The field-collected samples were kept fresh in a cool-box with an ice pack and then transported to the laboratory, where the specimens were pressed on herbarium sheets while still alive. Samples for morphological observation were preserved in 5% formalin/seawater. Voucher specimens were deposited in the herbarium of Jeju National University (JNUB, Jeju, Korea). California specimens borrowed from the University Herbarium, University of California at Berkeley (UC) were also examined (Figure 1, Table S1).

### 5.2. Classifying Specimens with Phenotype

All the specimens from the NW Pacific were divided into four phenotypes based on key characteristics outlined by Yoshida [23], including thallus width, branching pattern, the shape of the apex, and hook-formation: (1) *A. ciliolatum*-type (AC: subdichotomously branching linear thalli with hook-forming acute apices); (2) *A. flabellatum*-type (AF: flabellate thallus with a stipe, tapering branches, and slightly curved acute apices); (3) *C. hayamensis*-type (CH: di- to trichotomously branching linear thalli without hooks); (4) *C. membranacea*-type (CM: stipitate, broad, and palmate thallus with visible macro- and invisible microscopic veins and round apices without terminal hooks) (Figure 3). Two to five individuals with each phenotype and with overlapping phenotypes were chosen for molecular analyses (Figure 2, Figure 3 and Table S1).

### 5.3. Molecular Analysis

Samples for genomic DNA extraction were taken from selected pressed specimens. Total genomic DNA was extracted from the specimens and prepared with a DNeasy Plant Mini kit (Qiagen, Hilden, Germany), following the manufacturer’s protocol. The amplification for *rbc*L was conducted using the primers F7 (or F145), R898, F762, and R1442 [33,56]. Polymerase chain reaction (PCR) amplifications were performed in Swift MaxPro thermal cyclers (ESCO, Singapore) with an AccuPower PCR PreMix (Bioneer, Dajeon, Korea). Amplification reactions were set up and run as described in Saunders and Moore [57]. The PCR products were purified with an AccuPrep PCR Purification Kit (Bioneer, Daejeon, Korea) and sequenced commercially by Macrogen (Seoul, Korea). The electropherogram output of each sample was edited using Chromas Lite 2.01 (Technelysium, Helensvale, Queensland, Australia) and PHYDIT version 3.1 software [58]. The DNA sequences of *rbc*L were assembled in BioEdit 7.0.5 [59] and visually aligned. A total of 47 *rbc*L sequences were successfully amplified, including 29 from the NW Pacific, six from California, five from Spain, and seven from Tasmania and New Zealand (Table S1). The *rbc*L sequences in GenBank identified as *A. ciliolatum* from Chile (MH649426) and *A. venulosum* (AF254156) from UK were also aligned with sequences from our material. Because six *rbc*L sequences from Chilean samples in GenBank were all identical, we used only one sequence (MH649426) in our phylogenetic analysis (Figure 1 and Figure 2). The phylogenetic relationships of the 49 aligned *rbc*L sequences were investigated by comparing them with 15 taxonomic groups in GenBank, including ten assigned to the tribe Cryptopleureae and five to Neuroglosseae (as an outgroup).

The uncorrected pair-wise genetic distances (p) among the populations were estimated using the phylogenetic analysis software MEGA 4.0 [60] to assess the genetic variation of *rbc*L. Phylogenetic analyses were performed using RAxML [61], with the GTR + Γ + I evolutionary model. To identify the best tree, we constructed 200 independent tree inferences, using the -# option with the default -I (automatically optimized subtree pruning–regrafting rearrangement) and -c (25 distinct rate categories) software options. To generate bootstrap values, we used the same program with the same settings for 1000 replications. 

### 5.4. Morphological Observation

Sections for morphological examination were cut by hand using a razor blade, or with a bench-top freezing microtome (MFS no. 222; Nippon Optical Works, Tokyo, Japan). Sections and whole mount materials were either stained with 1% aniline blue acidified with 1% HCl and mounted in 40% corn syrup solution, or stained with Wittmann’s [62] aceto-iron-hematoxylin-chloral hydrate and mounted in 50% Hoyer’s mounting medium [41]. Images of the pressed specimens were captured using a G7x digital camera (Canon, Tokyo, Japan) and photomicrographs were taken using a BX 43 microscope (Olympus, Tokyo, Japan) with an EOS 600 D digital camera (Canon, Tokyo, Japan). The digitized images were imported into Adobe Photoshop (ver. 6.1) and edited to produce plates.

## Data Availability

Not applicable.

## Supplementary Materials

The following are available online at https://www.mdpi.com/article/10.3390/plants10112269/s1, Table S1: List of *Acrosorium* samples with collection information, phenotype, and GenBank accession numbers. Phenotype = AC, *Acrosorium ciliolatum*; AF, A. *flabellatum*; CH, *Cryptopleura hayamensis*; CM, *C. membranacea*.
